# Marked variation in predicted and observed variability of tandem repeat loci across the human genome

**DOI:** 10.1186/1471-2164-9-175

**Published:** 2008-04-16

**Authors:** Colm T O'Dushlaine, Denis C Shields

**Affiliations:** 1 Molecular and Cellular Therapeutics, Royal College of Surgeons in Ireland, Dublin 2, Ireland; 2 UCD Conway Institute of Biomolecular and Biomedical Research, University College Dublin, Belfield, Dublin 4, Ireland

## Abstract

**Background:**

Tandem repeat (TR) variants in the human genome play key roles in a number of diseases. However, current models predicting variability are based on limited training sets. We conducted a systematic analysis of TRs of unit lengths 2–12 nucleotides in Whole Genome Shotgun (WGS) sequences to define the extent of variation of 209,214 unique repeat loci throughout the genome.

**Results:**

We applied a multivariate statistical model to predict TR variability. Predicted heterozygosity correlated with heterozygosity in the CEPH polymorphism database (correlation ρ = 0.29, p < 0.0005) better than the correlation between the CEPH and WGS data (ρ = 0.17), presumably because the model smoothes noise from small sample sizes. A multivariate logistic model of 8 parameters accounted for 36% of the variation in the WGS data. Validation studies of 70 experimentally investigated TRs revealed high concordance with the model's predictions (p < 0.0001).

**Conclusion:**

Variability among 2–12-mer TRs in the genome can be modeled by a few parameters, which do not markedly differ according to unit length, consistent with a common mechanism for the generation of variability among such TRs. Analysis of the distributions of observed and predicted variants across the genome showed a general concordance, indicating that the repeat variation dataset does not exhibit strong regional ascertainment biases. This revealed a deficit of variant repeats in chromosomes 19 and Y – likely to reflect a reduction in 2-mer repeats in the former and a reduced level of recombination in the latter – and excesses in chromosomes 6, 13, 20 and 21.

## Background

Initial approaches to characterising variable number tandem repeats (VNTRs) in the human genome focussed on their utility as informative markers for gene mapping. However, the growing body of evidence that a subset of VNTRs play important roles in determining mammalian disease [1-3] and functional variation [4,5] has renewed interest in functional studies of VNTRs themselves, particularly where they occur in protein coding regions [6,7] or in promoters of genes [8-11]. VNTRs have a relatively high mutation rate, so that they may contribute to a substantially greater degree of genetic variation than point mutation changes, or indels in general. Recently, a large-scale assessment of insertion and deletion (indel) variation in the human genome has been conducted [12]. Of the total of 415,436 unique indels polymorphisms identified in this study, 122,458 (29.5%) were classified as repeat expansion polymorphisms. This study highlights the large amount of repeat variation that can be observed using sequence traces. However, it is not clear how biased these apparent findings are. False negatives arise from inadequate and biased sampling of chromosomes for sequencing (approximately 4.5 fold coverage on average, Lander et al. 2001), while false positives arise from genome assembly artefacts and sequencing errors. Here, we set out to determine whether the large repository of human shotgun genomic sequences could be interrogated to provide a useful predictive model for mammalian VNTR variation. Such a model would advance our understanding of the determinants of TR polymorphism, and provide a useful tool for the prediction of polymorphic TRs in mammals, that can guide experimental investigations.

A number of workers have developed relatively simple statistical models to predict which tandem repeats are likely to be variable. However, these have mostly relied on relatively small training datasets. Naslund and co-workers [13] developed a logistic regression model for predicting repeat variability, trained using 59 VNTRs and 56 monomorphic repeats, derived from the literature and from lab experiments. Similarly, Wren and colleagues developed a prediction tool, albeit a simpler one, derived from rules defined in the literature [14]. They experimentally confirmed the predictive accuracy of this tool, reporting an average polymorphism accuracy of 67% across 75 loci. An additional study compared two draft human genome sequences, the Human Genome Project (HGP) and Celera draft, to predict polymorphism (Denoeud et al. 2003). This was an attractive approach, particularly given that the two samples are being drawn from highly curated drafts of the human genome. Specifically, a criterion was described that was positive if the HGP and Celera drafts agreed on the length of a microsatellite and negative if they disagreed. The HGP and Celera drafts agreed in 65% of cases, but there is a substantial lack of independence arising from data-sharing during assembly. Secondly, for the 35% of repeats that differed in length between assemblies, 12% (5 of 41) of the human genome project sequence did not match any allele size identified by genotyping experiments and 76% (31 of 41) of the Celera DNA did not match. It was concluded that the Celera draft in particular was more prone to assembly error for the microsatellites examined in this study. For these reasons, we focussed in this study on the WGS sequences from HGP: one feature of this is that sequences are in their pre-assembled state, avoiding certain artefacts introduced during the assembly process.

Here, we applied and assessed a multivariate statistical model based on WGS data to predict tandem repeat variability in the human genome.

## Methods

### Detecting and assembling repeat variability data

Sequence files corresponding to human WGS data were downloaded from the Ensembl trace server [15]. A total of 32 GB of sequence data was downloaded. This was reduced to 4.9 GB by pre-screening all sequences with the Tandem Repeats Finder (TRF) program [16] and only retaining sequences containing tandem repeats. The coordinates for all known human genes were downloaded from the UCSC genome browser [17]. A file containing 2–12 mer tandem repeats detected by TRF under default settings (chromTrf.zip) from all chromosomes was also downloaded from this resource. The human genome release version used was hg17 (or NCBI build 35) (May 2004).

For each chromosome, gene coordinates and repeat coordinates and details were recorded. The chromosome was parsed to obtain flanking sequence information for repeats, 25 bases on each side. For each repeat, the flanks and their reverse-complement were searched against the WGS dataset and the length of the returned hit sequence was recorded. To minimise spurious hits due to sequence error, only hits where both flanks were perfectly matched to the target sequence were retained. All hits were checked to ensure that the length of the hit was consistent with a change in the copy-number of the reference repeat. Detected variants were screened to ensure that they represented length variation arising as copy-number differences in genomic DNA, rather than intron retention, alternative splicing or sequence errors, as follows: only length variations that corresponded to a length difference that was a multiple of the repeat unit were selected. For this set, tandem repeats were detected in the variant sequence and checked to ensure that the observed copy-number was in agreement with the expected one, given the length of the hit block and the length of the tandem repeat unit as follows:

For cases where the length of the hit tandem array is greater than that of the reference, we calculate *I*, a measure of the inconsistency of the variant with any multiple of the repeat copy number. This is calculated following [18]), and serves to describe how closely a length variant matches the expected lengths seen from changes that represent precise changes in copy-number. This option represents a stringent search for tandem repeat variation as we were specifically interested in investigating repeat variations consisting of multiples of the repeat unit and not other types of tandem repeat variations. In addition, it represents a check to ensure that the observed variant does not represent an alternative form of variation, for instance if the repeat was overlapping an alternatively spliced exon, or variation arising from sequence error.

WGS hits were defined as a "population" of repeats and the gene diversity (or heterozygosity) of this population was calculated as

$$H_{E}=1-\sum_{i=1}^{k}Pi^{2}$$

where *Pi *is the frequency of the *i*th of *k *repeat lengths at a locus [19]. For this analysis, only TRs of estimated unit lengths 2–12 were included.

### Mapping to CEPH

Mapping of repeat variants from the CEPH genotype database [20] to repeat variations detected from WGS sequences was implemented as follows: Firstly, a list of markers from the CEPH database version 10 (mkr.dbV10.all) were obtained from the CEPH website [21]. All D-numbers for markers, where available, were recorded. Secondly, STS (Sequence Tagged Site) markers corresponding to CEPH entries were obtained from UniSTS by searching the D-numbers from CEPH against the UniSTS human subset of UniSTS, a database of STSs unique to the human genome [22]. Finally, all STS markers obtained were searched against the sequences exhibiting tandem repeat sequence lengths variation within WGS sequences using e-PCR [23]. Successful matches could thus be directly compared to CEPH as a plot of heterozygosity of repeats obtained by the WGS method versus that of repeats existing in CEPH.

### Derivation of statistics describing repeats and their flanking sequences

Statistics for the repeat sequence were obtained from Tandem Repeats Finder [16]. Dinucleotide bias [24] was calculated for the repeat sequence and also for 20 bases of sequence flanking the repeat.

Programs from the EMBOSS suite [25] were used to obtain a number of additional variables describing sequences flanking repeats. The first is the melting temperature (Tm) for DNA which is computed by the DAN algorithm and based on experimentally-derived DNA nearest neighbour base pairing statistics [26]. (Tm is the temperature at which double-stranded DNA separates into two single strands.) The second is the fraction of bases 'G' and 'C' in the sequence. The third is the number of CpG motifs in the sequence. The latter two are known to be implicated in the mutability of a sequence with CpG motifs occurring on average once every 64 base pairs and mutate at a rate that is ten times higher than that of other single base sites [27,28]. As polymorphisms can be used to infer functional constraint [29], which might correlate with repeat variability, SNP information for sequences flanking repeats was also added from the HapMap database [30]; two variables were generated; the total number of SNPs within 1000 bp of flanking sequences (left and right combined) and the mean minor allele frequency for these SNPs. The minimum free energy of the estimated RNA secondary structure of the repeat sequence was also calculated, using the RNAfold program [31].

A final set of variables added were statistics on neighbouring functionally important regions of the genome. This is the distance from 3 functionally important and genetically distinct features of the human genome. The first of these was distance from the nearest promoter. Promoter coordinates were obtained from a large-scale study detecting 10,567 promoter regions in the human genome [32]. The second was distance from the nearest gene. The gene coordinates of 43,232 human genes were obtained from the KnownGene track of the UCSC genome browser [17]. The final set of features consisted of 699,647 human regions conserved between human, mouse and rat genomes (Multi-species Conserved Regions (MCSs)) [33]. As MCSs were derived from the most conserved subset of sequences from a 3-species sequence alignment, they served as a surrogate for sequence conservation, which can be an indication of functional importance/evolutionary constraint. This MCS dataset does not overlap known human coding regions and thus represents a different source of putative functionally important regions.

### Statistical modeling of repeat variants

Analysis was carried out in STATA using forward stepwise regression with a significance threshold of 0.01. Logistic regression modelling was used with the binary variable "yesnovar", describing whether or not the repeat was observed to be variant in the WGS dataset as the dependent variable, and a number of variables describing the repeat sequence and its flanks as potential predictor variables. The variable "pop_size" – the number of hits obtained from the WGS scan – was used to weight the data, thus giving greater weight to repeats with more hits, and an upper "pop_size" limit of 12 was imposed to limit the contributions of a small number of repeats (6%) with more than this number of hits in the WGS search as it is unlikely that sequences from more than 12 individuals overlap at a given repeat locus. These options were chosen on the basis that such modeling helped to maximize the % variance accounted for by the predictors.

To estimate the efficiency of unit-length specific models versus a generic model, their predictions were amalgamated in a "combined" model [see Additional file 1]. This was defined as a prediction for each repeat in the generic model that is obtained from one of the length-specific models; for all dimers, a prediction for the "combined" model is taken from the dimer-specific model, and so on.

## Results and discussion

### Detected repeat variations in the Whole Genome Shotgun (WGS) dataset

A total of 224,499 population sets with WGS hits were observed after searching 257,256 repeats against the WGS data. Of these, 215,147 remained after filtering out repeat variations inconsistent with a change in repeat copy-number. This number was reduced to 209,214 when repeats matching more than one region of the genome were excluded [see Additional file 2]. Variants were recorded where the variation was consistent with a copy-number change. A summary of detected variability among different length classes of repeats is shown in Table 1 and we also present the distribution of calculated heterozygosity values [see Additional file 3]. Repeats of longer unit length have a lower percentage of variants and on average about 51% of repeats examined had at least one detectable variant in the WGS dataset.

**Table 1:** Summary of variants and non-variants detected in the WGS dataset divided according to unit length of the repeat.

***Repeat class***	***Variants (%)***	***Non-variants (%)***	***Mean population size***	***s.d. population size***	***Mean Het*,**	***s.d. Het*.**
**2-mers**	58686 (76.2)	18369 (23.8)	5.53	6.05	0.40	0.26
**3-mers**	6928 (55.0)	5676 (45.0)	5.65	4.81	0.27	0.27
**4-mers**	27413 (54.1)	23299 (45.9)	5.71	5.05	0.27	0.27
						
**5-mers**	7478 (35.5)	13603 (64.5)	5.76	4.67	0.17	0.24
**6-mers**	2338 (21.3)	8638 (78.7)	5.66	6.66	0.09	0.19
**7–12 mers**	3400 (9.2)	33386 (90.8)	7.64	158.18	0.04	0.13

**Overall**	106243 (50.8)	102971 (49.2)	5.98	66.53	0.26	0.28

The levels of detected repeat variation seen in TRs of different length (Table 1), are in good agreement with a previous description of 45,411 TR indel variants in the human genome [12]. Of the 24,571 repeats reported in their study to be variant that were also reported in our analysis, 92% were reported as variant in our dataset.

For the 5,738 VNTRs with a unit length of greater than 6, there is an existing prediction method [13] applicable to VNTRs of this length. It predicted 24% of these to be variant (taking a cut-off of > = 0.5 on their normalised regression score); it predicted that 97.6% of the 42,024 invariant repeats were indeed invariant. This suggests that the Naslund method trained on a small dataset may tend to under-predict VNTRs, when using the 0.5 cut-off.

### Do the heterozygosity levels of the observed VNTRs in the WGS dataset correlate with those in the CEPH data resource?

How do we know the observations of repeat variation are real and not just an artefact of sequence error? The strategy to only report variants where the length difference was consistent with a change in repeat unit copy-number greatly reduces the number of putative false-positive variants. We compared our findings to an existing resource of genotyped repeat variants. The CEPH (Centre d'Etude de Polymorphism Humaine) dataset was chosen for this comparison [20]. 3,641 loci were compared between the two datasets. The mean heterozygosity in CEPH was 0.696 and in WGS sequences was 0.506. The correlation coefficient was relatively low (0.172) but significant (Spearman rank correlation, p < 0.0005). As it was likely that small WGS loci with very small population size increase noise in the data, the correlation was repeated comparing the 1,429 loci with greater than 5 sequences in the WGS dataset (N = 1429). With this subset, the correlation was 0.211 and again significant (p < 0.0005). The smaller WGS sample size, exacerbated by the non-independent re-sampling of the same WGS alleles for sequencing, may result in the slightly reduced heterozygosity for the WGS data versus the CEPH data.

### Predicting whether a TR is polymorphic or not

**Figure 1:**
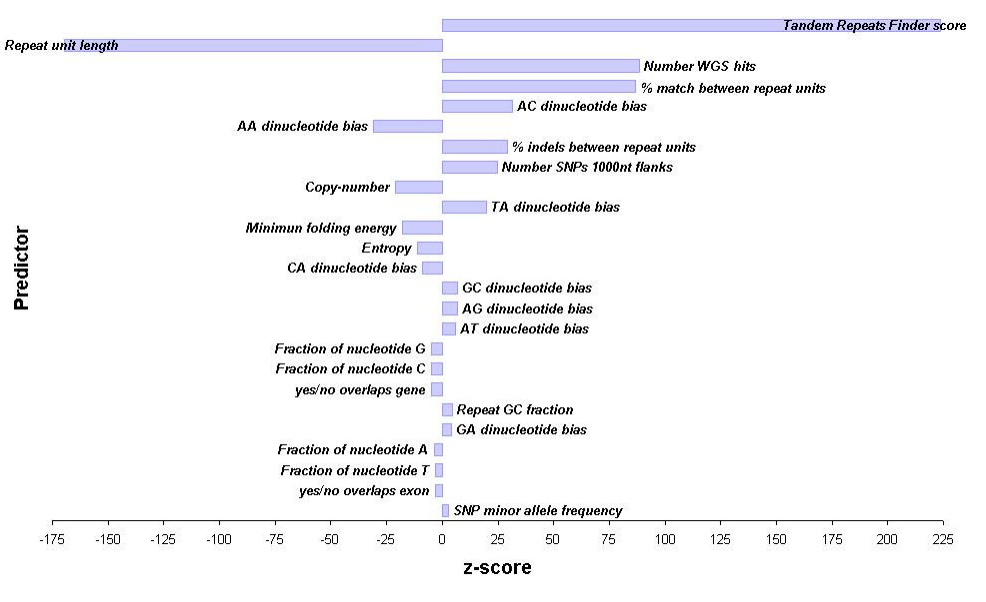
Significant predictors of repeat variability from the generic logistical model, sorted by absolute value of the z-score.

To determine if the polymorphism of a TR may be predicted from features of the sequence, we fitted univariate and multivariate logistic models of predictors to the WGS dataset. Predictors that were examined in this analysis are presented in Table 2. Our objective was to identify the major predictors of repeat variability, and present a predictive model that combined these factors. Various factors appear to be predictive when considered independently, and these are ranked in Table 3 in order of the degree to which they predict whether or not a TR is polymorphic. However many of these predictors are themselves strongly correlated. This makes it hard to distinguish independent and covarying predictors of TR polymorphism. Therefore, we concentrated our analysis on stepwise multivariate modelling. The important predictors in the multivariate model, and their directions of effects, are summarised in Figure 1. In both the univariate and the multivariate analysis, the unit length, the TRF score, the % match (Table 3 and Figure 1) are strong predictors. However, in other respects the results of the two analyses differ.

**Table 2:** Predictor variables derived from the repeat and flanking sequences whose impact on variability were tested by regression.

**Predictor name**	**Description**
**Statistics About The Tandem Repeat**	

**pop_size**	Population size: number of unique sequences from which estimate of repeat variability was obtained
**entropy**	Based on percentage composition: ∑ ω * log(*ω*) where *ω *is A, C, G and T. This is 0 when the repeat array consists of only one nucleotide and 2 when all nucleotides are equal [16]
**T**	%T in the repeat, e.g. 50% in TGTGTGTG
**G**	%G in the repeat, e.g. 50% in TGTGTGTG
**C**	%C in the repeat, e.g. 50% in CACACACA
**A**	%A in the repeat, e.g. 50% in CACACACA
**score**	Tandem Repeat Finder (TRF) [16] program-derived overall score
**%indels**	inferred consensus [16]
**%match**	% matches between actual repeat units and the inferred consensus [16]
**unit_length**	Length of tandem repeat unit, e.g. 2 for TGTGTGTG
**blocklength**	Length of the tandem repeat array, e.g. 8 for TGTGTGTG
**copy_number**	Number of copies of repeat unit, e.g. 4 for TGTGTGTG
**CG, CA, AC, AG, GA, CC, AT, TA, GC, AA**	Observed/expected dinucleotide bias of 10 dimers in the tandem repeat array [24]
**tm_repeat**	Melting temperature of the sequence [26]
**G+C_repeat**	Fraction of the sequence represented by the bases G or C, e.g. 0.5 for TGTGTGTG
**RNA_free_energy**	Free energy of the tandem repeat sequence RNA secondary structure [37]

**Statistics From 20 bp/500 bp Flanks of the Repeat**	

**tm_flank20, tm_flank500**	Melting temperature of the sequence [26]
**G+C_flank20, G+C_flank500**	Fractional G+C content of the two 20 bp/500 bp flanking sequences
**CpG_flank20, CpG_flank500**	Number of CpG (CG) dinucleotides in the two 20 bp/500 bp flanking sequences
**num_SNPs**	Total number of SNPs in the two 500 bp flanks
**SNP_allele_freq**	Mean SNP minor allele frequency for SNPs in the two 500 bp flanks

**Statistics on distance to nearest neighbouring elements**	

**nearest_promoter**	Distance in nucleotides from the nearest promoter
**nearest_gene/CDS**	Distance in nucleotides from the nearest gene/CDS
**nearest_MCS**	Distance in nucleotides from the nearest Multi-species Conserved Sequences defined in [33]
**nearest_CpG**	Distance in nucleotides from the nearest CpG island (defined by the UCSC genome browser)
**nearest_regulatory**	Distance in nucleotides from the nearest regulatory region (UCSC genome browser)

**Table 3:** Univariate logistic predictors of whether or not a TR is polymorphic in the Whole Genome Shotgun datasets.

**Predictor^1^**	***Variable class***	***Mean among variants***	***Mean among invariants***	***% increase among variants***	***Logistic Pseudo R*^2^**
unit_length	Repeat	3.115	5.828	-46.6	0.19
score	Repeat	84.569	61.891	36.6	0.14
copy_number	Repeat	20.440	11.724	74.3	0.08
%match	Repeat	94.261	89.711	5.1	0.05
AC	Repeat	2.252	1.396	61.3	0.04
%indels	Repeat	2.083	4.619	-54.9	0.04
CA	Repeat	2.280	1.757	29.8	0.03
SNP_allele_freq	Repeat flank	0.166	0.167	-0.6	0.02
AA	Repeat	1.025	1.183	-13.4	0.01
blocklength	Repeat	57.130	48.430	18.0	0.01
G+C_repeat	Repeat	32.098	28.592	12.3	< 0.01
entropy	Repeat	1.050	1.100	-4.5	< 0.01
CC	Repeat	1.052	1.150	-8.5	< 0.01
CpG_flank500	Repeat flank	4.695	5.496	-14.6	< 0.01
G+C_flank500	Repeat flank	41.427	42.363	-2.2	< 0.01
tm_flank500	Repeat flank	53.014	53.418	-0.8	< 0.01
G+C_flank20	Repeat flank	38.653	39.873	-3.1	< 0.01
tm_flank20	Repeat flank	51.606	52.134	-1.0	< 0.01
C	Repeat	15.695	13.831	13.5	< 0.01
G	Repeat	15.559	13.717	13.4	< 0.01
GC	Repeat	1.010	1.088	-7.2	< 0.01
TA	Repeat	0.921	0.877	5.0	< 0.01
A	Repeat	33.180	35.523	-6.6	< 0.01
CpG flank 20	Repeat flank	0.190	0.226	-15.9	< 0.01
tm_repeat	Repeat	48.744	47.967	1.6	< 0.01
num_SNPs	Repeat flank	2.380	2.220	7.2	< 0.01
RNA_free_energy	Repeat	-3.978	-3.157	26.0	< 0.01
nearest_promoter	Distant repeat flank	475996.5	439298.5	8.3	< 0.01
CG	Repeat	0.930	0.973	-4.4	< 0.01
AT	Repeat	0.991	0.963	2.9	< 0.01
AG	Repeat	1.313	1.354	-3.0	< 0.01
T	Repeat	34.591	35.767	-3.3	< 0.01
pop_size		6.317	5.642	12.0	< 0.01
nearest_gene	Distant repeat flank	143738.9	133874.5	7.4	< 0.01
nearest_CDS	Distant repeat flank	151516.1	141369.5	7.2	< 0.01
GA	Repeat	1.346	1.382	-2.7	< 0.01
nearest_regulatory	Distant repeat flank	-3642189	-3363138	8.3	< 0.01
nearest_MCS	Distant repeat flank	66672.9	72695.2	-8.3	< 0.01

^1 ^Significant with p < 0.00005 in both Mann-Whitney and t-test. Only variables with at least one significant p-value at the 5% level are shown, and dinucleotide biases in the flanking sequences of repeats were also excluded.

Though many predictors contribute very little to the overall model fit, a number of predictors stand out as very significant determinants (Figure 1). Restricting the multivariate model to the 8 most important parameters accounted for 36% of the variation, while adding other parameters to the model only marginally increased the variance accounted for. The AC dinucleotide bias in this model is mainly attributable to effects seen in a dimer TR model [see Additional file 4]. It is not too surprising that this effect is seen in the general model, given that AC repeats represent 25% of all dimers, and 10% of all repeats in the dataset. The increased mutability of AC dimers suggested by this model may in part explain why they have come to such a high frequency in the human genome, if there is also a bias towards insertional mutations at AC dimers. Two less important parameters in the model are the % of insertion/deletions, which increases variation, and the copy-number, which decreases variation. At first glance, this makes no sense whatsoever. Indeed, when considered on their own, they have the opposite effect (Table 3). This emphasizes the fact that these parameters are correlated with the score: the additional predictive information they provide here is against this background. Thus, the predictive model must be interpreted as a whole, and the directions of effects not taken out of context.

The three parameters that contributed most in determining variability were tandem repeat finder (TRF) program score, shorter unit lengths, and % match between TR units (accounting for 35% of the variation (Table 4)). Since the score is proportional to copy-number, these observations are consistent with previous models of TR variation (e.g. Wren et al. 2000). As most of the predictive potential of our model is captured by these three variables, researchers wishing to apply a simple version of our model to their own data may use the coefficients presented in Table 4, implemented in a simple PERL script originally developed by Naslund et al. (2005) [see Additional file 5]. This fits the following equation: predicted variability = 0.05*score - 0.452*unit_length + 0.066*%match - 7.585.

**Table 4:** Multivariate Analysis. Logistic regression coefficients for the 3 most predictive covariates are shown. A more detailed table, using all covariates, is given [see Additional file 6].

**Variable**	**Coefficient**	**S.E.**	**z-score**	**p-value**	**Confidence intervals**
Score	0.050	< 0.001	294.85	< 0.0001	0.0492083 → 0.0498669
Unit length	-0.452	0.001	-311.89	< 0.0001	-0.4545942 → -0.4489163
%match	0.066	< 0.001	196.10	< 0.0001	0.0657407 → 0.0670681
*intercept*	-7.585	0.035	-214.63	< 0.0001	-7.654186 → -7.515656

A more complete summary of models using all covariates is presented [see Additional file 6].

It is of interest to note that the number of SNPs within 1000 bases is associated with increased variability. This is consistent with the idea that certain regions of the human genome have higher overall variability than others, in large measure due to different population histories (e.g. selective sweeps, random drift), and possibly also due to a general increase in mutation of both SNPs and TRs in certain regions. However, this association was rather small, and given that it is rather negligible when considered in a univariate analysis (Table 3), we do not believe it is appropriate to over-interpret this data, especially given the non-independence of the determination of SNP density and TR variability, which are both influenced by the WGS sequences available.

The population size is a factor not strictly related to the repeat but rather to the amount of observations made of the repeat in the WGS data – it affects the likelihood of observing a variant but doesn't offer information about the repeat itself. Therefore, application of this model to sequence data for predictive purposes requires ignoring this parameter in the model. Therefore, the model serves primarily to rank, rather than provide a real prediction of the actual level of heterozygosity, or the actual probability that the sequence is variant (see also below).

### Do unit length specific models predict better than the generic logistic model?

To assess this, we fitted unit length specific models [see Additional file 4], and then compared the overall dataset with predictions that were drawn from a model specific to their unit-length, with the predictions that were provided from the generic model.

Here, the performance of the model was tracked by systematically varying the threshold for separating variants from invariants from 0 to 1 in increments of 0.01. Based on a given threshold, two groups are defined – predicted variants and predicted non-variants. These are then tabulated against the actual status (yes/no variant) of the repeat, forming a contingency table. From this contingency table, the true positive and true negative fractions can be estimated. The Receiver Operating Characteristic (ROC) curve derived from this data [see Additional file 1] indicates that the model representing the combination of length-specific models performs approximately the same as the generic model. We were somewhat surprised by this result, assuming in advance that certain unit lengths might have very specific predictive factors (such as the AC bias among dimers) that would substantially alter the model. This finding suggests that such differences are only relatively minor.

### Model assessment: training versus validation datasets

One means of evaluating the performance of the statistical models is to split the data in half, derive the model from one half of the data and test the model on the other half. We randomly split the data into two groups and the generic linear regression algorithm was applied to one half. Linear predictions based on this model were then made for all repeats in the second group and these predictions were compared to the observed WGS heterozygosity data. This process was repeated 100 times. The mean Spearman correlation (p < 0.0005 in all cases) for this comparison was 0.65, s.d. 0.002, comparing to the same value of 0.65 when no such splitting was performed. Thus, according to this comparison, the derived model does not appear to suffer from any obvious problem of over-fitting, which typically results from too few samples and/or too many parameters in the model.

### Comparisons of model predictions to observed data

**Figure 2:**
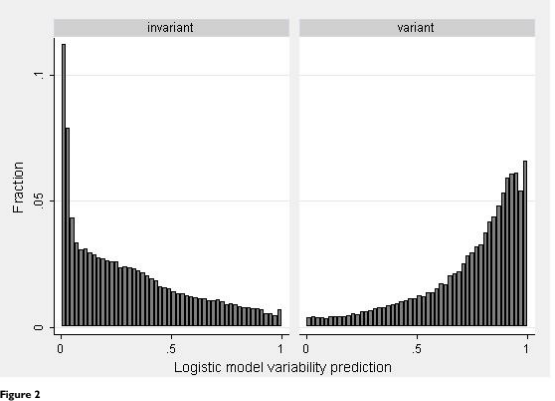
Histogram of predictions from the generic logistic regression model, broken down according to whether or not the repeats were variable.

Firstly, we compared the predictions of the multivariate logistic model to the observations within the WGS dataset itself. The distributions of predictions from the multivariate logistic model, divided across observed variant and invariant repeats are presented in Figure 2. We note that the correlations between heterozygosity observed in the WGS scan and heterozygosity predicted by the regression model are affected by sample size. For example, when only one hit is observed for a repeat (N = 36,822), the Spearman correlation is 0.55 (p < 0.0005) whereas it is in excess of 0.7 (p < 0.0005) when more than 4 hits are observed. Thus, we can be less confident in reported variants when the sample size is limited.

Secondly, we compared our results to repeats described by Naslund and colleagues [13]. This consisted of a collection of 59 polymorphic and 56 monomorphic repeats derived from both literature research and the laboratory genotyping of repeats. For 70 of these, a TR could be predicted by TRF, of which 30 were variant, and 40 invariant. 27/30 (90%) of their variants were also reported as such by our model and 29/40 (73%) were reported as invariant by our model, a highly significant correlation (Fisher's exact test p < 0.0001).

Thirdly, we compared our results to predictions made by the method described by Wren and colleagues, (2000). For variant repeats reported by the WGS search that were also 100% homogenous (N = 53,726), 99.76% were also predicted by the Wren et al. method to be variant.

### Heterozygosity prediction

To determine predictors of heterozygosity, forward stepwise linear regression was carried out. It is of interest to compare the prediction of heterozygosity from the linear regression, to the observed values inferred from the dataset. The correlation between observed heterozygosity and the predictions was 0.65 (Spearman), p < 0.0005.

The heterozygosity predictions from the linear regression can also be compared to real genotypic data. While the correlation between the observed CEPH heterozygosity and the observed WGS heterozygosity for matched TRs was 0.172 (Spearman, p < 0.0005), the correlation between the observed CEPH heterozygosity and the predicted heterozygosity from the generic linear regression model was 0.291 (p < 0.0005). This implies that an investigator interested in ranking the likely variability of human TRs would be typically better off using the predictions from the model, than interrogating the WGS data directly. This may be because the model smoothes over the noise due to small sample sizes for many of the WGS populations. It is likely that this conclusion applies not only to the linear regression model that predicts heterozygosity levels, but also to the logistic model predicting whether or not a TR is polymorphic. We caution that the heterozygosity calculation is prone to high levels of variability because sample sizes are often limited: even though its distribution achieves normality on average [see Additional file 3], only 30,193 of variant repeats (28% of all variant repeats) have more than 5 sequences supporting the existence of a variant (i.e. popsize > = 6).

### Genomic distributions

**Figure 3:**
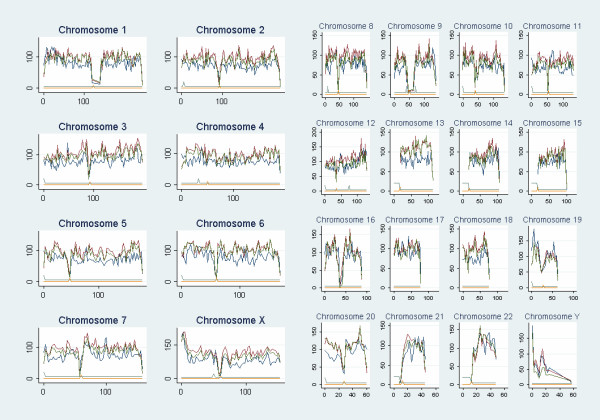
**Distributions of predicted invariant (blue), predicted variant (red) and observed variant repeats (green) across the human genome.** Counts are shown as counts of repeats per 2.5 Mb (y-axis) against genomic position in Mb (x-axis). Gap regions (light blue) and centromeres (orange) are also highlighted as peaks or lines raised above the base level. These regions were not included in the frequency estimations.

The distributions of repeats, observed and predicted VNTRs ("observed"= observed VNTR; "variant" = predicted VNTR, "invariant"= predicted invariant) were analysed in 250 Mb bins on a per chromosome basis (Figure 3). We noted a deficit of variant repeats compared to invariant repeats on the Y chromosome (mean ratio 0.64, s.d. +/- 1.02) [see Additional file 7], an observation that is not simply a result of reduced sample size [see Additional file 8]. There is also the suggestion that a similar relative deficit exists for chromosome 19 (0.78 +/- 0.15) [see Additional file 7]. How can this overall deficit be explained? We inspected the distribution of variant and invariant repeats along the chromosomes. Along the Y chromosome, there is considerable regional variation in repeats (Figure 3), with the variant and invariant repeats showing a similar pattern of distribution. This suggests that there may be a general depression of mutation to VNTRs, perhaps reflecting the lack of recombination, which may promote VNTR generation. The p terminus of Y does not show this deficit, perhaps in part due to the influence of recombination with X in the pseudoautosomal region. Along chromosome 19, there was a marked excess of invariant repeats compared to variant repeats at certain locations, particularly marked near the p terminus. The variability of the variant/invariant ratio among regions within a chromosome is most marked for Chromosome 21 [see Additional file 7]. This reflects a higher proportion of predicted variant repeats between the centromere and half way along the long arm, compared to the remained of the long arm.

Some chromosomes also appear to have excesses of variant repeats relative to invariant ones [see Additional file 7], particularly chromosomes 6 (1.27 +/- 0.24), 13 (1.35 +/- 0.29), 20 (1.25 +/- 0.31) and 21 (1.26 +/- 1.02). For each chromosome, we analysed the fraction of total repeats on that chromosome that constituted repeats of unit length 2–6 and found no evidence suggesting that biases in the chromosomal distributions of different unit length repeats might explain the excesses highlighted above [see Additional file 9]. However, we did observe that a lower proportion of repeats on chromosome 19 were 2-mers: 2-mers constituted 27% of all repeats on chromosome 19, compared to an average of 44% across the genome. This may explain the reduction in variable repeats on this chromosome, as 2-mers are more prone to polymorphism [14], with a mean heterozygosity of 0.40 compared to an average over all repeats of 0.26.

## Conclusion

Our goal was to develop a predictive model of TR variability based on WGS data. Linear models accounted for roughly one third of the observed variance [see Additional file 4]. The general model over all unit lengths (from 2 to 12) appears to function remarkably well, in comparison with unit-length specific models. This observation suggests that the underlying mutation processes that generate, expand and contract TRs may be similar for all 2–12 mer repeats, since the quantifiable factors that predict variation are shared.

The use of whole-genome shotgun sequences to identify repeat variations has been shown to be possible. Clearly, there are a number of limitations and inaccuracies arising from the use of shotgun sequence data that affect the results. These range from the rate of sequence error to the lack of a defined population sampling for different regions. Since the predictive model that we developed here in fact correlates better with CEPH TR heterozygosity levels, we conclude that the best practise in identifying variant repeats from the human genome may be to rely on the predictive model, rather than relying on interrogation of the WGS database.

Analysis of the distributions of variant and invariant repeats across chromosomes revealed certain chromosomes with excesses (6, 13, 20, 21) or deficits (19, Y) of variant repeats. For the Y chromosome, reduced levels of recombination may help to explain the observed deficit. The link between microsatellite variability and recombination rate in humans has been investigated [34] but no strong evidence was found. Therefore, background selection is unlikely to be the major factor affecting the disparate ratios of variant to invariant repeats across chromosomes: mutation rate variation, divergent levels of different repeat types across chromosomes and locus-specific effects, such as selection, are more likely.

This work represents a considerable step towards quantifying the predictive power of a number of sequence characteristics in relation to repeat polymorphism. It also provides estimates of the extent of variation of 209,214 unique repeat loci throughout the genome [see Additional file 2] providing a framework for guiding repeat selection in studies such as case-control studies of repeat variants, in combination with the predictive models generated here. We anticipate that knowledge gleaned from this work will assist in the selection of optimal candidate repeats for future genotyping experiments and in the identification of a greater number of unstable or polymorphic tandem repeats with potentially significant functional effects. Observations made in this study may also be applied to other mammalian species for the *de novo *prediction of repeat variability. We provide a PERL script that implements the logistic model presented here, in addition to models described by Wren and colleagues and Naslund and colleagues [see Additional file 5]. The script may also be downloaded from our website [35]. In addition, we provide a custom track on the UCSC genome browser [36]. Alternatively, interested researchers can upload data we provide [see Additional file 10] as a custom track to UCSC. This track also provides information about the tandem repeat and its variability. Repeat ids are provided on this track and may be linked with data we provide [see Additional table 1a-d] which provides more detailed information on the nature of the repeat and its variability.

## List of abbreviations

CEPH: Centre d'Etude du Polymorphisme Humain; HGP: Human Genome Project; ROC: Receiver Operating Characteristic; SNP: Single Nucleotide Polymorphism; STS: Sequence Tagged Site; TR: Tandem repeat; TRF: Tandem Repeats Finder; VNTR: Variable Number of Tandem Repeat; WGS: Whole-genome Shotgun.

## Authors' contributions

CO'D carried out the analysis and primary manuscript writing. DS participated in study design, provided intellectual input and contributed to manuscript writing. All authors read and approved the final manuscript.

## Supplementary Material

Additional file 1 ROC curves illustrating the behaviour of the different models. Each point corresponds to a threshold dividing predictions from the model into variants or non-variants. These predictions were then compared to the original WGS estimate of repeat variability. "Generic" represents predictions from the model trained on all the data. "Exonic" represents the model trained on repeats only within exons. "Combined" represents predictions taken for each repeat that were derived from a specific model for that repeat, i.e. for all dimer repeats, the prediction from a model trained on all dimers in the entire dataset was taken. These length-specific models were derived for 2-,3-,4-,5-,6- and 7–12 mer repeats and then combined. Click here for file

Additional file 2 209,214 repeats searched against WGS sequences. Data is presented for chromosome, start position, stop position, sequence of the tandem repeat unit, repeat unit copy-number, repeat block length, number of unique repeat block lengths, heterozygosity of the repeat, the number of times each unique repeat block length arises from the search against the WGS sequences, unique repeat id. Click here for file

Additional file 3 Distribution of heterozygosity for repeats searched against the WGS dataset. Click here for file

Additional file 4 Summaries of the different stepwise logistic and linear regression models tested when modelling all covariates. The Pseudo R^2 ^and R^2 ^are used here as estimates of model fit. For all models, *popsize *is used to weight the data and only repeats with *popsize *< = 12 are modelled. Click here for file

Additional file 5 PPR script. Distribution of program developed to predict potentially polymorphic. repeats. Click here for file

Additional file 6 Summary of models using all covariates. Click here for file

Additional file 7 Mean ratio of variant to invariant repeats over all chromosomes. Standard deviations from this mean (calculated in windows of 250 Mb) are shown as error bars. Click here for file

Additional file 8 Genomic distribution of density of different repeat types and of mean popsize. Density of A repeats, B variant repeats and C predicted variants over all chromosomes. Density is calculated as the sum total of non-gapped, non-telomeric sequence divided by the number of observations for each repeat type and is thus lower when more observations are made. The distribution of mean popsize (D) is also shown. Click here for file

Additional file 9 The fraction of different length repeats per chromosome. For each chromosome, the fraction is the count of each repeat type divided by the total number of 2–6-mer repeats on that chromosome. Click here for file

Additional file 10 UCSC browser custom track information for detected tandem repeat variants. Click here for file
