# Supplementary figures and images for: Marked variation in predicted and observed variability of tandem repeat loci across the human genome
Source: BMC Genomics. 2008 Apr 16;9:175. doi: 10.1186/1471-2164-9-175 (PMC2364633; doi:10.1186/1471-2164-9-175)

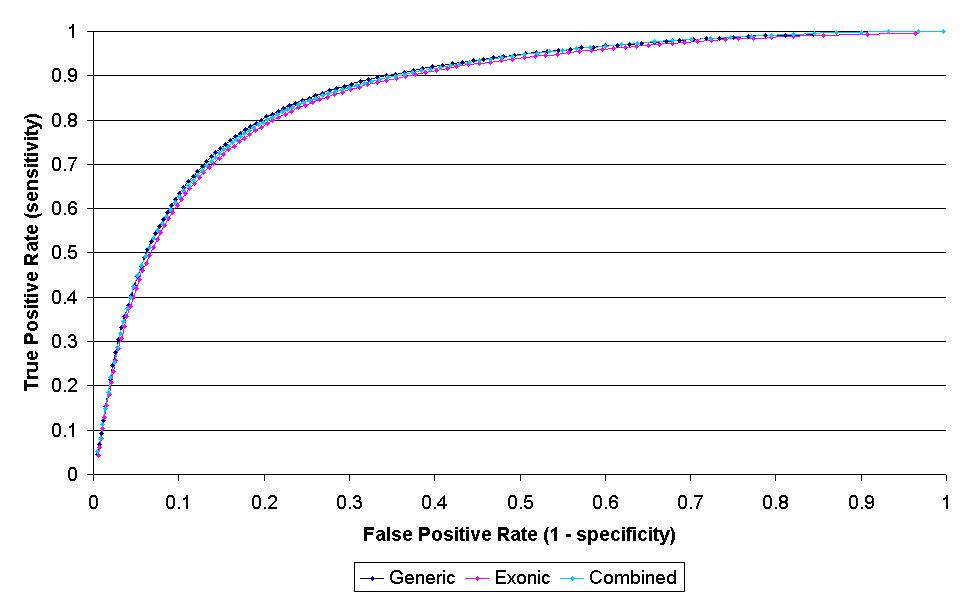

Supplement: Additional file 1 — ROC curves illustrating the behaviour of the different models. Each point corresponds to a threshold dividing predictions from the model into variants or non-variants. These predictions were then compared to the original WGS estimate of repeat variability. "Generic" represents predictions from the model trained on all the data. "Exonic" represents the model trained on repeats only within exons. "Combined" represents predictions taken for each repeat that were derived from a specific model for that repeat, i.e. for all dimer repeats, the prediction from a model trained on all dimers in the entire dataset was taken. These length-specific models were derived for 2-,3-,4-,5-,6- and 7–12 mer repeats and then combined. [file 1471-2164-9-175-S1.tiff]

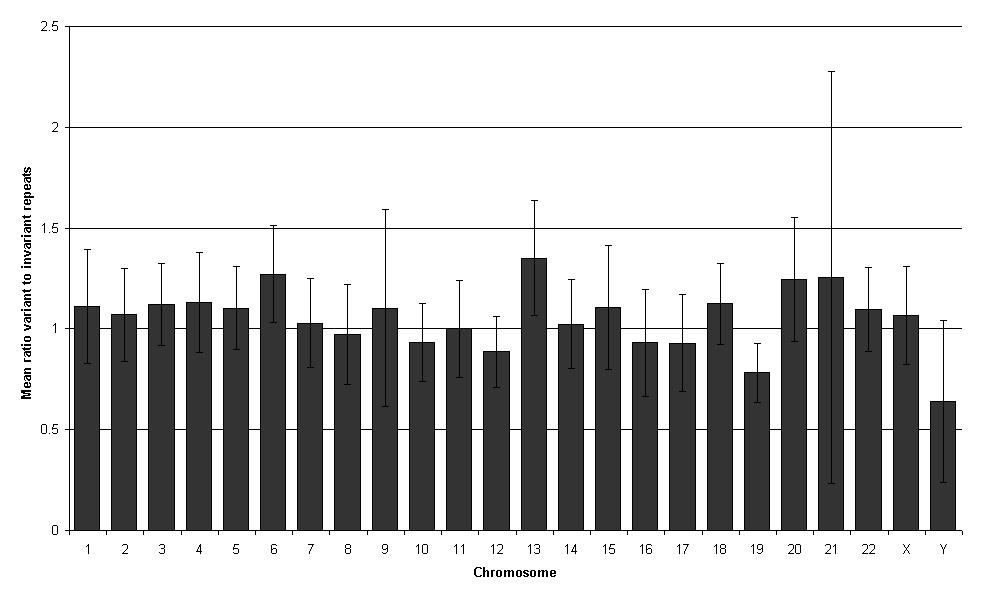

Supplement: Additional file 7 — Mean ratio of variant to invariant repeats over all chromosomes. Standard deviations from this mean (calculated in windows of 250 Mb) are shown as error bars. [file 1471-2164-9-175-S7.tiff]

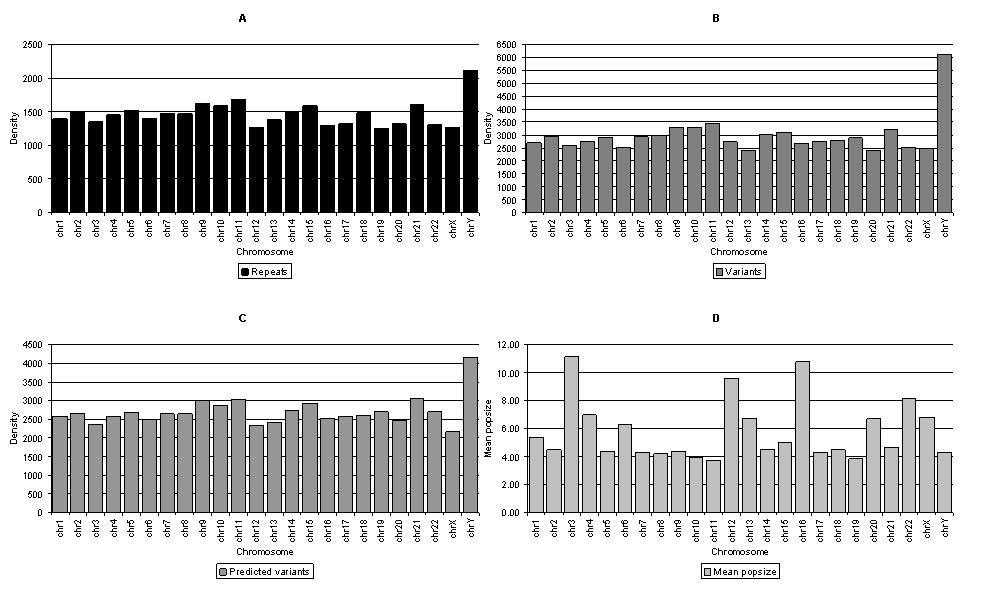

Supplement: Additional file 8 — Genomic distribution of density of different repeat types and of mean popsize. Density of A repeats, B variant repeats and C predicted variants over all chromosomes. Density is calculated as the sum total of non-gapped, non-telomeric sequence divided by the number of observations for each repeat type and is thus lower when more observations are made. The distribution of mean popsize (D) is also shown. [file 1471-2164-9-175-S8.tiff]

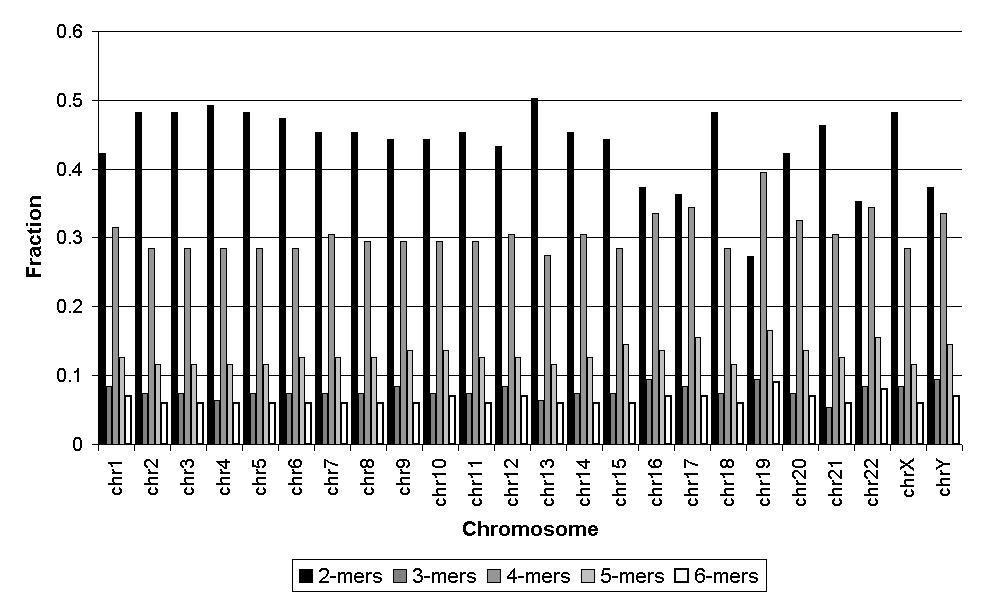

Supplement: Additional file 9 — The fraction of different length repeats per chromosome. For each chromosome, the fraction is the count of each repeat type divided by the total number of 2–6-mer repeats on that chromosome. [file 1471-2164-9-175-S9.tiff]
