# Supplementary material for: Marked variation in predicted and observed variability of tandem repeat loci across the human genome
Source: BMC Genomics. 2008 Apr 16;9:175. doi: 10.1186/1471-2164-9-175 (PMC2364633; doi:10.1186/1471-2164-9-175)
Supplement: Additional file 4 — Summaries of the different stepwise logistic and linear regression models tested when modelling all covariates. The Pseudo R2 and R2 are used here as estimates of model fit. For all models, popsize is used to weight the data and only repeats with popsize < = 12 are modelled. [file 1471-2164-9-175-S4.doc]

Additional File 4

| ***Model*** | ***Adjusted***  ***Pseudo R^2^***  ***(logistic regression)*** | ***Adjusted***  ***R^2^***  ***(linear regression)*** | ***N***  ***variant/invariant***  ***% variant*** |
| --- | --- | --- | --- |
| Generic model | 0.3705 | 0.4209 | 209214  106243/102971  50.78 |
| 2-mer specific model | 0.1927 | 0.2378 | 77055  58686/18369  76.16 |
| 3-mer specific model | 0.2607 | 0.3225 | 12604  6928/5676  54.97 |
| 4-mer specific model | 0.2551 | 0.3195 | 50712  27413/23299  54.06 |
| 5-mer specific model | 0.2624 | 0.3429 | 21081  7478/13603  35.47 |
| 6-mer specific model | 0.1366 | 0.1531 | 10976  2338/8638  21.30 |
| Other (7- to 12-mers) | 0.1663 | 0.1807 | 36786  3400/33386  9.24 |
| Exon specific model* | 0.3944 | 0.4630 | 2101  701/1400  50.07 |

*only repeats occurring within exonic regions of genes are considered in this model.
