# Supplementary material for: Marked variation in predicted and observed variability of tandem repeat loci across the human genome
Source: BMC Genomics. 2008 Apr 16;9:175. doi: 10.1186/1471-2164-9-175 (PMC2364633; doi:10.1186/1471-2164-9-175)
Supplement: Additional file 6 — Summary of models using all covariates. [file 1471-2164-9-175-S6.doc]

Additional file 6

| ***predictor*** | ***generic***  ***logistic***  ***coeff.*** ^1^ | ***generic***  ***logistic***  ***z*** ^2^ | ***generic***  ***linear***  ***coeff.*** | ***generic***  ***linear***  ***t*** ^3^ | ***2-mer***  ***coeff.*** ^4^ | ***2-mer***  ***z*** | ***3-mer***  ***coeff.*** | ***3-mer***  ***z*** | ***4-mer***  ***coeff.*** | ***4-mer***  ***z*** | ***5-mer***  ***coeff.*** | ***5-mer***  ***z*** | ***6-mer***  ***coeff.*** | ***6-mer***  ***z*** | ***7-12***  ***-mer***  ***coeff.*** | ***7-12***  ***-mer***  ***z*** | ***exon***  ***coeff.*** | ***exon***  ***z*** |
| --- | --- | --- | --- | --- | --- | --- | --- | --- | --- | --- | --- | --- | --- | --- | --- | --- | --- | --- |
| score | 0.05 | 223.77 | 0.00 | 301.15 | 0.05 | 113.00 | 0.07 | 49.63 | 0.05 | 103.22 | 0.07 | 65.51 | 0.06 | 39.02 | 0.03 | 52.08 | 0.05 | 19.45 |
| unitlen | -0.44 | -169.75 | -0.03 | -245.66 | NA ^5^ | NA | NA | NA | NA | NA | NA | NA | NA | NA | -0.19 | -25.69 | -0.45 | -25.03 |
| popsize | 0.11 | 88.39 | 0.01 | 58.38 | 0.15 | 63.87 | 0.11 | 23.84 | 0.11 | 48.70 | 0.11 | 29.89 | 0.10 | 17.76 | 0.09 | 20.09 | 0.10 | 7.08 |
| pcmatch | 0.07 | 86.63 | 0.01 | 152.52 | 0.12 | 69.30 | 0.10 | 28.92 | 0.09 | 53.09 | 0.07 | 19.93 | 0.01 | 2.71 | -0.03 | -22.33 | 0.07 | 10.22 |
| ac | 0.29 | 31.53 | 0.04 | 61.16 | 0.12 | 23.02 | -0.16 | -5.64 | NA | NA | NA | NA | 0.10 | 3.52 | NA | NA | 0.18 | 5.16 |
| aa | -0.29 | -30.78 | -0.05 | -73.72 | -0.14 | -6.15 | 0.31 | 6.43 | NA | NA | 0.21 | 6.10 | 0.15 | 3.22 | 0.15 | 5.16 | NA | NA |
| pcindels | 0.03 | 29.17 | 0.00 | 43.39 | 0.02 | 10.60 | 0.05 | 9.25 | 0.04 | 16.34 | 0.02 | 4.87 | NA | NA | NA | NA | 0.05 | 4.14 |
| num_snps1000 | 0.04 | 24.80 | 0.00 | 29.16 | 0.04 | 14.86 | 0.05 | 8.76 | 0.04 | 15.21 | 0.06 | 12.73 | NA | NA | 0.03 | 5.24 | NA | NA |
| copynum | -0.01 | -21.23 | 0.00 | -8.20 | NA | NA | -0.02 | -7.33 | -0.01 | -5.47 | 0.03 | 5.39 | -0.03 | -5.54 | NA | NA | NA | NA |
| ta | 0.18 | 19.79 | 0.03 | 36.36 | NA | NA | 0.12 | 2.84 | 0.19 | 11.25 | NA | NA | 0.26 | 6.72 | 0.10 | 4.19 | NA | NA |
| rnafold | -0.01 | -17.75 | 0.00 | -4.42 | NA | NA | NA | NA | 0.02 | 4.99 | -0.03 | -6.47 | -0.02 | -5.62 | -0.01 | -10.16 | 0.02 | 4.61 |
| _cons | -7.24 | -11.81 | -0.74 | -129.36 | -15.22 | -67.45 | 0.93 | 0.33 | -1.06 | -0.92 | -13.22 | -34.49 | -6.29 | -22.98 | 13.72 | 7.60 | -10.08 | -13.82 |
| entropy | -0.25 | -11.31 | NA | NA | 0.97 | 13.65 | -0.70 | -6.50 | -0.19 | -4.77 | -0.28 | -3.59 | -0.35 | -4.18 | -0.65 | -13.01 | 0.77 | 5.13 |
| ca | -0.09 | -9.05 | -0.02 | -21.73 | NA | NA | -0.17 | -5.84 | -0.09 | -7.86 | NA | NA | NA | NA | 0.11 | 5.34 | NA | NA |
| gc | 0.02 | 6.83 | 0.00 | 15.22 | 0.04 | 7.49 | NA | NA | 0.02 | 3.50 | NA | NA | NA | NA | NA | NA | -0.29 | -3.98 |
| ag | 0.07 | 6.82 | 0.00 | 4.38 | NA | NA | NA | NA | 0.22 | 14.55 | 0.16 | 5.27 | 0.23 | 5.12 | 0.23 | 8.52 | NA | NA |
| at | 0.05 | 5.90 | 0.01 | 11.63 | 0.13 | 7.44 | 0.16 | 4.20 | 0.06 | 4.50 | NA | NA | NA | NA | 0.23 | 10.59 | NA | NA |
| g | -0.03 | -4.82 | 0.00 | 23.35 | -0.05 | -18.86 | -0.11 | -3.91 | -0.10 | -8.15 | -0.03 | -4.84 | -0.01 | -5.67 | -0.14 | -7.41 | -0.01 | -3.57 |
| c | -0.03 | -4.74 | 0.00 | 22.36 | -0.05 | -18.95 | -0.11 | -3.83 | -0.09 | -8.05 | -0.03 | -4.69 | -0.01 | -6.53 | -0.14 | -7.64 | NA | NA |
| yesnogene | -0.04 | -4.70 | -0.01 | -6.66 | NA | NA | NA | NA | -0.06 | -3.56 | NA | NA | -0.20 | -4.61 | NA | NA | NA | NA |
| gc_repeatblk | 0.01 | 4.50 | NA | NA | 0.04 | 16.11 | -0.04 | -5.19 | -0.03 | -8.70 | 0.02 | 3.24 | NA | NA | NA | NA | NA | NA |
| ga | 0.04 | 4.02 | 0.01 | 11.56 | NA | NA | -0.13 | -4.96 | NA | NA | 0.12 | 3.60 | 0.24 | 5.49 | 0.07 | 2.70 | NA | NA |
| mean_gc_f~20 | 0.00 | -3.96 | 0.00 | -8.34 | 0.00 | 5.63 | NA | NA | 0.00 | 5.72 | 0.01 | 6.73 | NA | NA | -0.01 | -5.67 | NA | NA |
| a | -0.02 | -3.65 | NA | NA | NA | NA | -0.15 | -5.18 | -0.12 | -10.45 | 0.00 | -10.27 | -0.01 | -11.91 | -0.15 | -8.11 | NA | NA |
| t | -0.02 | -3.18 | 0.00 | 20.35 | NA | NA | -0.15 | -5.11 | -0.12 | -10.27 | NA | NA | NA | NA | -0.14 | -7.80 | NA | NA |
| yesnoexon | -0.12 | -3.12 | -0.02 | -5.37 | NA | NA | NA | NA | NA | NA | NA | NA | NA | NA | NA | NA | NA | NA |
| mean_mi~1000 | 0.08 | 2.77 | NA | NA | NA | NA | 0.58 | 5.15 | NA | NA | NA | NA | NA | NA | 0.57 | 5.61 | NA | NA |
| cg | NA | NA | NA | NA | NA | NA | 0.34 | 4.43 | NA | NA | NA | NA | NA | NA | NA | NA | 0.50 | 5.40 |
| yesnoregpot | NA | NA | 0.00 | 3.81 | NA | NA | NA | NA | NA | NA | NA | NA | NA | NA | -0.18 | -5.58 | NA | NA |
| cc | NA | NA | NA | NA | NA | NA | NA | NA | 0.03 | 3.66 | NA | NA | NA | NA | -0.04 | -3.04 | NA | NA |
| mean_cpg_~20 | NA | NA | -0.01 | -10.74 | -0.12 | -8.29 | NA | NA | NA | NA | 0.14 | 4.86 | 0.11 | 2.93 | 0.18 | 5.65 | NA | NA |
| yesnocpg | NA | NA | -0.02 | -2.71 | 1.17 | 2.61 | -0.51 | -2.58 | -2.27 | -3.73 | NA | NA | 1.27 | 5.44 | NA | NA | NA | NA |

^1^ The coefficient is the value for the regression equation for predicting the dependant variable (heterozygosity for linear regression, yes or no variant for logistic regression) from the independent variable (the variable in question)

^2^ The logistic regression z value, used to test the null hypothesis that there is no significant difference in the distribution of a predictor variable between variants and non-variants. A coefficient is assigned to each predictor in the model and the higher it is, the greater the predictive power of that predictor. The coefficient estimate has a standard error and the z-score represents the coefficient normalised by the standard error of that coefficient

^3^ The linear regression t value, used to test the null hypothesis that there is no significant difference in the dependence of a predictor variable on the dependant variable, in this case heterozygosity

^4^ This and subsequent models are logistic regression models of specific subsets of the data, in this case modelling of dimers only. In the case of the ‘exon’ model, this represents the modelling of repeats in exons only. Equivalent linear regression models of these subsets give very similar results and therefore are not presented

^5^ ‘NA’ indicates that the predictor variable was not a significant predictor under this model
